# Evolution of small putative group I introns in the SSU rRNA gene locus of *Phialophora *species

**DOI:** 10.1186/1756-0500-4-258

**Published:** 2011-07-22

**Authors:** Lorena B Harris, Scott O Rogers

**Affiliations:** 1 Department of Biological Sciences, Bowling Green State University, Bowling Green, OH 43403, USA; 2 Department of Biology University of South Florida, 4202 E. Fowler Avenue, SCA110, Tampa, Florida 33602, USA

**Keywords:** Ribosomal RNA genes, Phialophora, Intron, group IC1

## Abstract

**Background:**

Group I introns (specifically subgroup IC1) are common in the nuclear ribosomal RNA genes of fungi. While most range in length from more than 200 to nearly 1800 nucleotides (nt) in length, several small putative (or degenerate) group I introns have been described that are between 56 and 81 nt. Although small, previously we demonstrated that the *Pa*SSU intron in the rRNA small subunit gene of *Phialophora americana *isolate Wang 1046 is capable of *in vitro *splicing using a standard group I intron pathway, thus qualifying it as a functional ribozyme.

**Findings:**

Here, we describe eight short putative group I introns, ranging in length from 63 to 75 nt, in the rRNA small subunit genes of *Phialophora *isolates, a fungal genus that ranges from saprobic to pathogenic on plants and animals. All contain putative pairing regions P1, P7, and P10, as well as a pairing region formed between the middle of the intron and part of the 3' exon. The other pairing regions common in the core of standard group I introns are absent. However, parts of the 3' exon may aid in the stabilization of these small introns. Although the eight putative group I introns were from at least three species of *Phialophora*, phylogenetic analysis indicated that the eight are monophyletic. They are also monophyletic with the small introns of two lichen-forming fungi, *Porpidia crustulata *and *Arthonia lapidicola*.

**Conclusions:**

The small putative group I introns in *Phialophora *have common features that may represent group I introns at their minima. They appear to have a single origin as indicated by their monophyly in phylogenetic analyses.

## Background

Group I introns are capable of self-splicing *in vitro*, and members of the IC1 subgroup are relatively common in the nuclear ribosomal RNA genes of fungi [1-3]. A few hypotheses have been proposed to explain how group I introns became established in the rRNA gene locus. Intron homing is one of the most likely mechanisms for converting an intron-less allele into one containing an intron, and has been demonstrated in experimental studies. This is initiated by the product of an intron-encoded homing endonuclease gene (HEG), which cleaves an intron-less allele at or near the intron insertion site [3-5]. This generates a double-stranded DNA break at the site of intron insertion. The intron-containing allele is used as the template to repair the break resulting in insertion of the DNA version of the intron and co-conversion of flanking exon sequences. However, very few group I introns contain open reading frames, indicating that the HEG's have been lost, they are elsewhere in the genome (and act in trans) or they are no longer functional in the genome rendering the introns immobile. Establishment of group I introns in the rRNA gene locus appears to have occurred tens to hundreds of millions of years ago, since the introns are found in phylogentically diverse organisms and the sequence diversity is large.

There are no known advantageous effects of group I introns to host organisms [6]. They have been reported as selfish or parasitic genes that are adapted to assure their survival. However, some studies suggest that their widespread distribution indicates their evolutionary success and importance [7-10]. The presence of these introns in a broad range of species is due to a dynamic equilibrium between gains, mutations, and losses tempered by maintenance of accurate splicing of the exons. Both vertical and horizontal transmissions have been demonstrated, and transposition has been proposed as the mechanism that is responsible for movement of the introns within genomes [3-5,9,11].

In previous studies we reported the presence of self-splicing group I introns in the rRNA small subunit (SSU) genes of *Cenococcum geophilum *[12], as well as the location and self-splicing ability of a small (67 nucleotides, nt) group I intron in *Phialophora americana *Wang 1046 [13,14]. This small intron may have originated from an unequal crossover in an ancestor that was amplified in the genome. Gene conversion, which is active in the rRNA gene locus, may have increased copy number of the mutant. Strong selective pressure is in effect in the rRNA locus to retain the production of the large number of rRNAs required in each cell. Therefore, the small introns must have retained their ability to splice in order to allow the production of functional small subunit rRNA. In this study we compared eight small putative group I introns from different isolates of *Phialophora *species (anamorphic dematiaceous fungi) in order to study the structure and evolution of these introns after their establishment in the rRNA genes. We show that the small introns differ in sequence, but retain similar structural features.

## Methods

*Phialophora *isolates (*P. americana *CDC 5, CDC 10, CDC B3733, NIH 8730, Wang 1046, Wang 10507, Wang 10508; *P. verrucosa *NIH 8701; and *P. europea *CDC B1214) were obtained from Dr. CJK Wang, State University of New York, College of Environmental Science and Forestry, Syracuse, New York. They had been isolated from plant and human sources (Table 1). All were grown on MEA (malt extract agar [1.28% maltose, 0.28% dextrin, 0.24% glycerol, 0.08% peptone, and 1.5% agar]) for 14 days at 22°C in 9-cm Petri dishes. Liquid cultures were grown in PDB (potato dextrose broth [0.4% potato starch and 2.0% dextrose]). Morphological analysis was performed to confirm taxonomic characters for the species by light, scanning electron and confocal laser scanning microscopy [15,16]

**Table 1:** List of isolates and sequences used in this study.

Species	Isolate	Intron Length (nt)	Accession Number	Source of Isolate
*Phialophora americana*	CDC 5	65	JF414780	*Tilia *sp., Virginia, USA, Conant 743
	CDC 10	65	JF414776	Paper pulp, Wisconsin, USA, Conant 333
	CDC B3733	0	na	Human foot lesion biopsy, Wisconsin, USA
	NIH 8730	67	JF414779	Unknown
	Wang 1046	67	JF414775	*Fraxinus *sp., New York, USA, DAOM 64689
	Wang 10507	66	JF414777	Decaying wood, New York, USA
	Wang 10508	63	JF414778	Decaying wood, New York, USA

*Phialophora europea*	CDC B1214	72	JF414782	Human

*Phialophora verrucosa*	NIH 8701	75	JF414781	Human, Texas, USA

**Figure 1:**
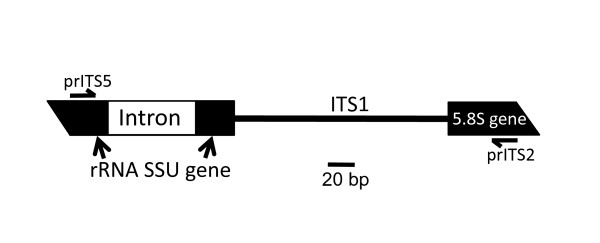
**Region of the rRNA gene locus that was characterized**. This fragment from all isolates was amplified by PCR with primers prITS2 and prITS5, and sequenced. It includes the 3' end of the SSU rRNA gene (33 bp on the 5' side of the intron), the intron (63-75 bp), the remainder of the SSU rRNA (22 bp), internal transcribed spacer 1 (ITS1), and the 5' end (50 bp) of the 5.8S rRNA gene.

DNA from mycelia was extracted using a CTAB (cetyltrimethylammonium bromide) preparation method [17-19]. The DNA was examined by electrophoresis at 5 V/cm for 1 h on 1% agarose gels in TBE (89 mM Tris-base, 89 mM borate, 2 mM EDTA, pH 8.0), containing 0.5 μg/ml ethidium bromide, and quantified by UV fluorescence intensity. Polymerase Chain Reaction (PCR) was performed to amplify fragments from 33 bp upstream of the intron site in the SSU rRNA gene through 50 bp of the 5' end of 5.8S rRNA gene (Figure 1). PCR was performed using 0.5 mM each of primer prITS2 (GCTGCGTTCTTCATCGATGC) and prITS5 (GGAAGTAAAGTCGTAACAAGG) [20] using a GenAmp PCR Reagent Kit (Applied Biosystems, Foster City, CA). A Thermal Controller (GeneAmp PCR System 9700, Applied Biosystems, Foster City, CA, USA) was used with a program consisting of 2 min at 94°C, followed by 35 cycles of: 1 min at 94°C, 2 min at 54°C, and 2 min at 72°C. This was followed by a final extension of 10 min at 72°C. Amplification was confirmed by subjecting aliquots to electrophoresis on 2% agarose gels (with TBE and ethidium bromide, as described above). The PCR products were purified using a QIAquick PCR purification kit following the manufacturer's instructions (QIAGEN, Valencia, CA). Both strands were submitted for sequence determination (Gene Gateway LLC, Hayward, CA) using primers prITS2 and prITS5.

Multiple sequence analyses were performed to corroborate the presence and position of the introns in the isolates. Intron sequences were used to compare among the isolates. Sequences were aligned using CLUSTALW2 http://www.ebi.ac.uk/Tools/clustalw2/index.html. The alignments were examined, and manual adjustments were performed. Phylogenetic analyses were performed with PAUP (Phylogenetic Analysis Using Parsimony; [21]) using Maximum Parsimony, with bootstrapping.

The intron sequences were subjected to secondary structure analysis using Mfold (vers. 2.2, [22]). Because of the short lengths of the introns, after the initial determinations of secondary structure, parts of the sequences were removed and reanalyzed separately. Final manual adjustments were made in each case.

## Results

**Figure 2:**
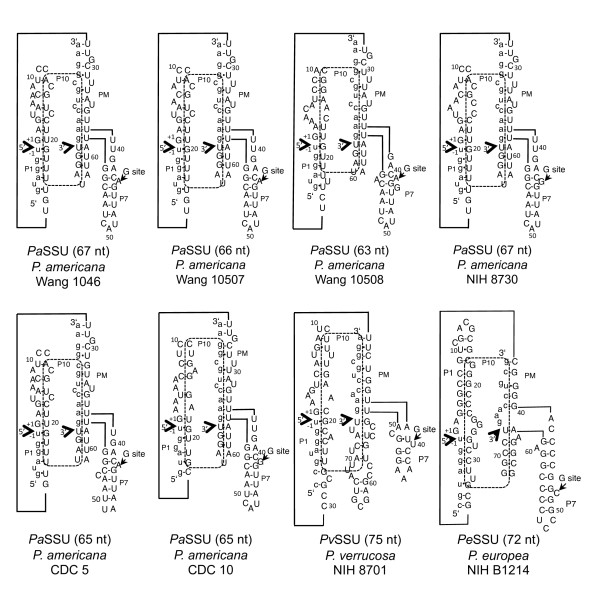
**Comparison of intron secondary structures**. The intron sequences are in upper case font, while the 5' and 3' exons are in lower case. Each of the introns includes a P1, P7 and P10 (formed by the 3' end of the intron and part of the 3' exon, paired with part of P1), as well as a pairing region between the middle portion of the intron and the 3' exon (labeled PM). The 5' and 3' splice sites are indicated by large arrowheads. The site that holds the guanosine that initiates the first trans-esterification reaction is indicated (G site with arrow).

**Figure 3:**
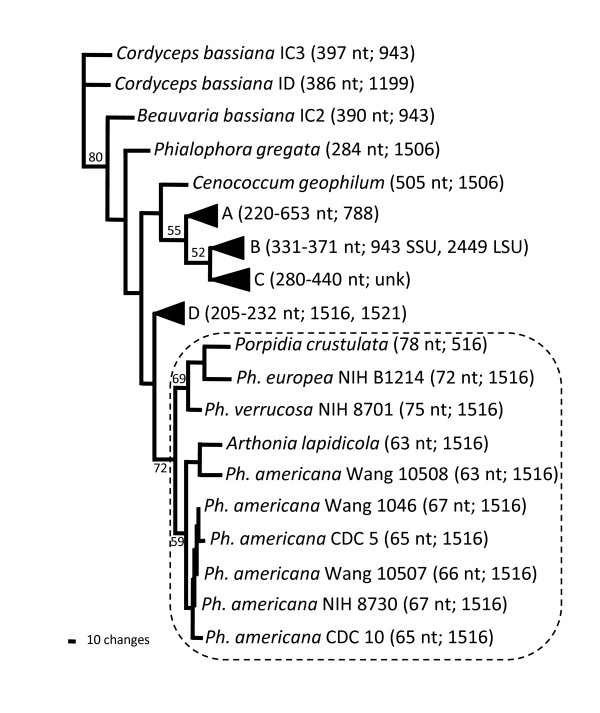
**Phylogenetic tree comparing the small putative group IC1 introns in the rRNA small subunit gene of species of *Phialophora *(*Ph*.), *Arthonia *and *Porpidia *with other group I introns found in the nuclear rRNA gene locus**. The tree presented is the single most parsimonious tree (determined using PAUP [21]), based on 115 nucleotide positions (only P1, P9, and the intron portion of P10 were used for the analyses). Gaps were counted as a fifth base. Bootstrap values (based on 1000 replications) greater than 50% are shown for the supported branches. The tree has 748 steps, with a consistency index of 0.5040. When gaps were considered as "missing data", 12 most parsimonious trees resulted, with 495 steps and a CI of 0.5515. While the branching pattern differed among the 12 trees, the small putative introns always formed a monophyletic clade. Triangles indicate clades that are summarized for simplicity, as indicated as follows: Clade A, subgroup IC1 introns from *Ascolacicola austriaca, Cryptendoxyla hypophloia*, and *Leucostoma personii*; Clade B, subgroup IC1 introns from *Gaeumannomyces graminis*, *Hypocrea pallida*, *Mycoarachis inversa*, and *Nectria aureofulva*; Clade C, subgroup IC1 introns from *Apioplagiostoma aceriferum*, *Cephalotheca sulfurea*, *Cordycepioideus bisporus*, and *Gnomoniella tubiformis; *Clade D, subgroup IC1 introns from *Ahtiana sphaerosporella*, *Arctocetraria nigricascens*, *Arctoparmelia centrifuga*, *Asahinea chrysantha*, *Cavernularia lophyrea*, *Cetraria ericetorum*, *Cetraria islandica *(3 isolates), *Cetraria nigricans*, *Cladonia arbuscula*, *Melanelia hepatizon*, *Melanelixia fulginosa*, *Myelochroa metarevuluta*, *Nephromopsis ahtii*, *Nephromopsis komarovii*, *Nephromopsis laureri*, *Nephromopsis stracheyi*, *Parmelia cochleata*, *Parmelia fertilis*, *Parmelia saxatilis*, *Parmelia squarrosa*, *Parmelia sulfata*, *Pseudophebe pubescens*, *Tuckermanella coralligera*, *Tuckermanella orbata*, *Tuckermanella playyphylla*, *Vulcipida pinasrti *and *Vulcipida viridis*. Accession numbers for the *Phialophora *sequences are provided in Table 1, while those for the other sequences are listed in reference [13]. Lengths of the introns are indicated within the parentheses, as are the physical positions in the rRNA genes. All locations are in the rRNA SSU gene, relative to the *E. coli *rRNA SSU gene, except where noted (i.e., 2449 LSU is in the rRNA LSU gene). Dashed box indicates the clade that includes all of the small putative group I introns compared in this study.

Previously, the small intron (*Pa*SSU) from *P. americana *Wang 1046 was demonstrated to splice *in vivo *[14], has group I ribozyme activity *in vitro*, and phylogenetically falls within the IC1 subgroup [13]. All of the isolates examined (except CDC B3733, which is intron-less) contain small introns (63-75 nt in length, designated *Pa*SSU, *Pv*SSU, or *Pe*SSU) located in the SSU rRNA gene at position 1516 (relative to *E. coli*) that can form similar secondary structures (Figure 2). Nucleotides characteristic of the *Pa*SSU for group I intron are found in all of the introns at the 5' and 3' splice sites. Because the *Pa*SSU intron from *P. americana *Wang 1046 has been demonstrated to splice *in vitro *via a group I intron mechanism, it is assumed that the small putative introns in the eight isolates exhibit similar splicing characteristics. Further evidence for this is provided by phylogenetic analysis, which indicates that the small putative introns form a monophyletic clade (Figure 3). Small degenerate introns from the lichen-forming fungi, *Porpidia crustulata *(78 nt intron) and *Arthonia lapidicola *(63 nt intron) also fall within the same clade with all of the *Phialophora *small putative introns, suggesting that this intron may be widespread among certain groups of fungi and might have been generated by a single mutational event.

**Figure 4:**
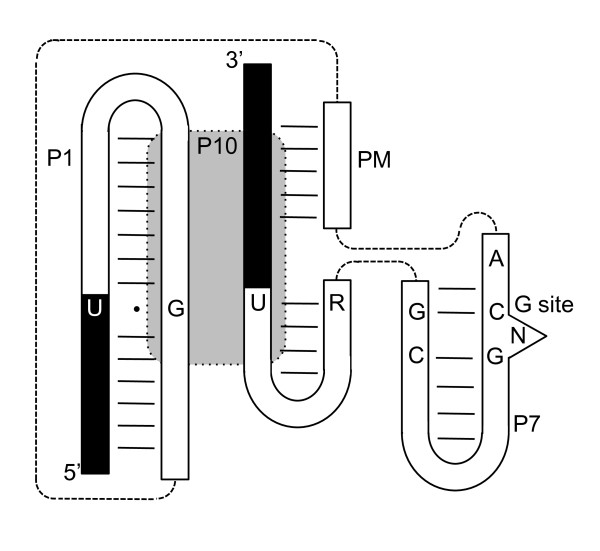
**Model of the small putative group I intron secondary structure based on conserved structures**. Exons are represented by black regions. Intron regions are in white. Dashed lines indicate regions connected within the intron. Conserved nucleotides are shown (although there are some G-C pairing differences in P7 in the introns from isolates NIH 8701 and NIH B1214). Grey area indicates P10. PM is the middle pairing region.

Secondary structures all were consistent and comparable (Figure 2), although some (e.g., *Pe*SSU and *Pv*SSU) varied to a greater extent in sequence, which is typical of group I ribozymes. All contained a G·U pair at the 5' splice site within the P1 pairing region, which consisted of part of the 5' exon, and the first 20-30 nucleotides of the intron (Figures 2 and 4). The mid-section of the intron paired with part of the 3' exon. All possessed a P7 that contained an unpaired nucleotide next to a G-C pair that holds an exogenous guanosine (characteristic of all group I ribozyme P7 regions), as well as an unpaired A on the 5' side of P7. Downstream from the P7 region, all had small paired regions that preceded the 3' splice site. This section also contained half of P10. The other half of P10 (the internal guide sequence, or IGS) was within P1, which is characteristic of group I introns. In isolate *P. americana *Wang 1046, previously we demonstrated [13] that the final nucleotide of the *Pa*SSU intron is a U (an ωU, instead of the common ωG in most other group I introns). Each of the other small introns also had a U that is similar to the one in isolate Wang 1046 at the 3' end of the intron (Figures 2 and 4).

## Discussion

The *Pa*SSU intron (67 nt in isolate Wang 1046), located in the rRNA SSU gene of *P. americana *(Figure 1), is the smallest known intron with demonstrated group I ribozyme activity [13]. Its size affords an opportunity to study the evolution of intron function. The mechanism of splicing differs slightly from that of standard group I introns. It proceeds through two trans-esterification reactions, as in the group I intron of *Tetrahymena thermophila *[23,24], but certain variations have been found in the second reaction, in that it employs a U (an omega ωU) as the last nucleotide of the intron instead of the canonical ωG found in almost all other group I introns investigated to date [13]. The other small introns described here are in the same location (nucleotide 1516, relative to *E. coli*) as the *Pa*SSU group I intron in isolate Wang 1046 (except the intron from *Porpidia crustulata*, which is at the 516 position [7]). While they all have similar secondary structure, their sequences differ.

Secondary structure conservation is evident in these small introns. Pairing regions P1, P7, and P10, as well as another pairing region between the middle of the introns and the 3' exons (termed PM) all were maintained in each of the introns (Figure 2). Furthermore, from our previous report [13], the P7 regions of these small introns probably originated as part of the P9.2 regions of larger group I introns. While the important secondary structures of these small introns are maintained, they alone may not be sufficient to hold the intron in the active tertiary structure for splicing to occur. Other parts of the rRNA gene locus may aid in this process. For example, in our previous *in vitro *splicing experiments, when RNA was used that included only the intron plus approximately 20 nt of each of the 5' and 3' exons, splicing was inefficient or failed. However, when a longer section (approximately 300 nt, including the entire ITS1) of the exon was included, splicing occurred *in vitro *[13]. Therefore, part of the SSU and ITS1 rRNA may form the tertiary structure that maintains splicing activity in these small putative group I introns.

Ribosomal RNA genes are arranged in tandem arrays of 60-200 copies in fungi. Recombination and gene conversion act to homogenize the individual repeats. However, mutations can lead to a heterogeneous combination of sequences. In one case, an intron-less allele coexisted with intron-containing versions. Sequence results demonstrated that there often were one or more mutant versions of the introns in each of the isolates (not shown). While it is unknown whether the mutant introns are functional, some differed from the functional *Pa*SSU intron in isolate Wang 1046, and therefore they may be pseudogenes. Vertical transmissions, mutations and losses are common in the evolution of group I introns in fungi [10,25]. The small putative introns in *Phialophora*, all appear to have originated from a single unequal crossover event that occurred in a common ancestor [13]. This is supported by three characteristics: 1. All are found at a single nucleotide position in the rRNA SSU gene, 2. All have similar secondary structures, and 3. They form a single monophyletic clade.

Many studies have shown evidence for stable maintenance of group I introns over long periods of time [1,26,27], which seems to be the case with the small putative introns described here [13]. Previous studies suggested the presence of group I introns sporadically in distant lineages of organisms that did not reflect the overall phylogeny. Those results suggest horizontal transmission between distinct lineages [3,11,28-30]. However, most of these involved large introns, the largest of which sometimes contained genes for homing endonucleases. No open reading frames that encode proteins for mobility such as homing endonucleases or reverse transcriptases have been reported in any *Phialophora *species. Certainly, a protein encoded from these small putative introns is implausible, and in general, open reading frames are absent from introns in the 1506-1521 location of the rRNA SSU genes in fungi. However, the different location for the *Porpidia crustulata *small degenerate intron (at position 516) suggests that it might have inserted into the rRNA locus through horizontal transfer or transposition.

Comparison of the *Pa*SSU (Wang 1046) intron with other introns inserted at the same site indicate that they are orthologs, although their sequences and lengths vary [1,12,13]. Group I introns inserted at different sites have been shown to be more distantly related even in the same species [3,10,31] and thus are likely to be paralogs. However, introns that are within the 1506-1521 region group together in phylogenetic analyses [13], and may represent a single insertion event, followed by mutation and use of cryptic splice sites. This creates the appearance of intron movement. However, the change in the splice site might not have been caused by translocation or transposition of the intron. The similarities in intron sequences among the isolates and the similarity to the other group I introns in the same region of the SSU rRNA gene, and the fact that they appear to be monophyletic [13] indicates that these introns moved into this location at a time that predated the separation of these taxa, which probably occurred at least tens of millions of years ago. Recombination, mutation, gene conversion, and vertical inheritance have resulted in the present distribution and diversity of group IC1 introns in the 1506-1521 region of the rRNA SSU genes in fungi, including the presence of these small putative group I introns.

## Conclusions

The set of small putative group I introns from the SSU rRNA genes of *Phialophora *species are monophyletic, presumably having originated from a single mutational event. All are similar to the functional putative group I intron from *P. americana *isolate Wang 1046, in that each possess a P1, P7 and P10. Although their primary sequences have diverged, they all have a G·U pair at the 5' splice site, a G-C pair in the P7 guanosine site, an unpaired A on the 5' side of P7, and a U as the final nucleotide of the intron. These introns may represent the minimum size for group I ribozymes.

## Competing interests

The authors declare that they have no competing interests.

## Authors' contributions

LH and SR designed the study, produced the secondary structure models, and contributed equally in writing the manuscript. LH performed all of the laboratory work and initial sequence analysis. All authors have read and approved the final manuscript.
